# Granulocyte heterogeneity in immune-mediated and inflammatory diseases: insights from single-cell transcriptomic analyses

**DOI:** 10.1093/intimm/dxag026

**Published:** 2026-05-26

**Authors:** Hiroshi Shimagami, Atsushi Kumanogoh, Masayuki Nishide

**Affiliations:** Department of Respiratory Medicine and Clinical Immunology, Graduate School of Medicine, The University of Osaka, Suita, Osaka 565-0871, Japan; Department of Immunopathology, World Premier International Research Center Initiative (WPI), Immunology Frontier Research Center (IFReC), The University of Osaka, Suita, Osaka 565-0871, Japan; Department of Advanced Clinical and Translational Immunology, Graduate School of Medicine, The University of Osaka, Suita, Osaka 565-0871, Japan; Department of Respiratory Medicine and Clinical Immunology, Graduate School of Medicine, The University of Osaka, Suita, Osaka 565-0871, Japan; Department of Immunopathology, World Premier International Research Center Initiative (WPI), Immunology Frontier Research Center (IFReC), The University of Osaka, Suita, Osaka 565-0871, Japan; Department of Advanced Clinical and Translational Immunology, Graduate School of Medicine, The University of Osaka, Suita, Osaka 565-0871, Japan; Integrated Frontier Research for Medical Science Division, Institute for Open and Transdisciplinary Research Initiatives (OTRI), The University of Osaka, Suita, Osaka 565-0871, Japan; Japan Agency for Medical Research and Development—Core Research for Evolutional Science and Technology (AMED–CREST), The University of Osaka, Suita, Osaka 565-0871, Japan; Center for Infectious Diseases for Education and Research (CiDER), The University of Osaka, Suita, Osaka 565-0871, Japan; Center for Advanced Modalities and DDS (CAMaD), The University of Osaka, Suita, Osaka 565-0871, Japan; Department of Respiratory Medicine and Clinical Immunology, Graduate School of Medicine, The University of Osaka, Suita, Osaka 565-0871, Japan; Department of Immunopathology, World Premier International Research Center Initiative (WPI), Immunology Frontier Research Center (IFReC), The University of Osaka, Suita, Osaka 565-0871, Japan; Department of Advanced Clinical and Translational Immunology, Graduate School of Medicine, The University of Osaka, Suita, Osaka 565-0871, Japan

**Keywords:** eosinophil, neutrophil, single-cell RNA sequencing, spatial transcriptomics

## Abstract

Neutrophils and eosinophils have long been regarded as terminal effectors of innate immunity. Technical advances in single-cell RNA sequencing define transcriptome-based granulocyte subsets beyond classical density- and surface marker-based classifications. These approaches reveal substantial heterogeneity in granulocyte differentiation states, activation programs, and tissue adaptation across human diseases. In microscopic polyangiitis, type II interferon (IFN) pathways shape a pathogenic neutrophil activation state, and serum IFNγ levels at disease onset may serve as a potential biomarker for subsequent relapse. In infectious diseases, including coronavirus disease 2019 (COVID-19) and sepsis, the expansion of immunosuppressive *ARG1*- and *IL1R2*-expressing neutrophils is reported in severe disease and may reflect altered immune responses. *MMP9*-high neutrophils are enriched in cardiovascular disorders and may be linked to thrombosis and ischemic injury. In allergic diseases, spatial and single-cell analyses identify tissue-specific eosinophil states and their interactions with epithelial and macrophage compartments, highlighting context-dependent eosinophil activation within inflamed tissues. Despite emerging evidence for disease-associated granulocyte heterogeneity, whether these populations causally contribute to disease pathophysiology remains largely unclear. Experimentally validated functions and clinically applicable surrogate markers, such as surface markers or circulating proteins, are still needed. This review summarizes recent advances and current limitations in understanding granulocyte heterogeneity across immune-mediated and inflammatory diseases and discusses how integrative single-cell approaches may support the development of clinically relevant biomarkers and targeted therapeutic strategies.

## Teaser

Granulocyte heterogeneity revealed by scRNA-seq

## Introduction

Human granulocytes are classified into neutrophils, eosinophils, and basophils. Of them, neutrophils are the most abundant white blood cells in human peripheral blood. Historically, neutrophils were not regarded as major regulators of adaptive immune responses; instead, they were considered “terminal effector cells” of innate immunity. Early studies focused on their role in phagocytosis and reactive oxygen species (ROS) production (1), which mediate the intracellular killing of bacteria (2). Further, neutrophil granules contain various antimicrobial proteins that are released upon degranulation and suppress bacterial growth (3, 4).

Over the past three decades, substantial heterogeneity has been revealed among human neutrophils, including differences in morphology, density, maturation status, surface protein expression, and potential roles in adaptive immune responses (5). Until the 1980s, mature neutrophils were believed to have a minimal capacity for *de novo* protein synthesis. Studies in the 1990s demonstrated that mature neutrophils are capable of producing and releasing cytokines and chemokines, including tumor necrosis factor (TNF) and C-X-C motif chemokine ligand 8 (CXCL8) (6, 7). These findings established neutrophils as terminal effectors as well as active regulators of inflammatory responses through the production of soluble mediators. In 2004, a major conceptual advance further expanded our understanding of neutrophil function. Human neutrophils release mesh-like structures composed of DNA, histones, and granule proteins, termed neutrophil extracellular traps (NETs) (8). NETs contribute to antimicrobial defense and have been implicated in a broad range of pathological conditions, including autoimmune diseases and thrombosis (9). Neutrophil function is not restricted to nonspecific innate immune responses. Under certain conditions, neutrophils express human leukocyte antigen class II (MHC-II) molecules and present antigens to T cells (10). Neutrophils also produce B-cell-activating factors and a proliferation-inducing ligand (APRIL), thereby contributing to B-cell maturation and potentially supporting antibody class switching and somatic hypermutation (11, 12). More recently, polymorphonuclear myeloid-derived suppressor cells (PMN-MDSCs) have been described as neutrophil-like cells with potent immunosuppressive activity. These cells express arginase 1 (ARG1) and inhibit T-cell responses (13).

To link functional diversity with phenotypic features, human neutrophils are divided into low-density neutrophils (LDNs) and normal-density neutrophils (NDNs) based on density-gradient centrifugation. Although LDNs exhibit enhanced NET formation (14), they also display immunosuppressive activity in infection and malignancy (15, 16). These data indicate that density alone is insufficient to define functionally distinct subsets of neutrophils. Efforts have also been made to classify neutrophils based on their surface protein expression levels. CD10-negative human neutrophils are morphologically immature and exhibit strong T-cell-activating capacity (17). In contrast, lectin-like oxidized low-density lipoprotein receptor-1–positive neutrophils display marked immunosuppressive activity, consistent with the PMN-MDSC phenotype (18). Despite these pioneering insights, no single marker can capture the full spectrum of neutrophil heterogeneity, making it challenging to establish specific neutrophil subsets as reliable biomarkers or therapeutic targets in disease conditions.

Single-cell RNA sequencing (scRNA-seq) is used to analyze gene expression in individual cells, allowing the detection of cell states that are difficult to distinguish using conventional approaches. scRNA-seq has been applied to human samples obtained from healthy individuals and patients with diverse diseases to provide insights into disease pathogenesis. Recently, scRNA-seq has enabled the refined dissection of granulocyte heterogeneity, linking distinct granulocyte subsets to disease states. Although cancer-associated granulocyte heterogeneity is a major area of single-cell research (19), granulocyte heterogeneity in immune-mediated and inflammatory diseases is a more recently emerging field that provides distinct insights into immune dysregulation, tissue inflammation, and relapse prediction. In this review, we summarize the technical advances in granulocyte scRNA-seq and discuss the clinical relevance of single-cell-based granulocyte profiles across disease contexts, focusing on autoimmune, autoinflammatory, allergic, and infectious diseases.

## Key technologies for scRNA-seq of granulocytes

It is technically challenging to analyze granulocytes by scRNA-seq because these cells generally contain relatively low amounts of RNA (20). Moreover, neutrophil activation and degranulation are highly sensitive to sample-processing conditions, including processing delays, temperature, density-gradient centrifugation, and red blood cell lysis (21). In eosinophils, high intracellular RNase activity may further compromise transcriptome profiling (22). Granulocytes are also susceptible to cell loss during cryopreservation and thawing (23). Therefore, freshly processed samples and optimized protocols for cell isolation and RNA capture are important for granulocyte scRNA-seq.

During the application of scRNA-seq to granulocytes, cell isolation and library preparation methods substantially influence cell recovery and data quality (24). Among widely used commercial platforms, the Chromium system (10x Genomics) relies on droplet-based encapsulation of single cells into gel bead-in-emulsion droplets for cell lysis and barcode tagging (25), whereas the Rhapsody system (BD Biosciences) captures individual cells with barcoded beads in microwell arrays (26). Microwell-based methods facilitate the detection of neutrophils compared with droplet-based methods (27). Comparative analyses indicate that microwell-based approaches may be better suited than droplet-based methods for detecting neutrophils, partly because they have been reported to capture a greater number of mRNA molecules per cell (28). Given the low RNA content of neutrophils, this higher transcript recovery may improve neutrophil detection and enable more comprehensive profiling of their transcriptional heterogeneity. However, given the rapid evolution of single-cell technologies, ongoing improvements in chemistry, sample handling, and RNA capture should be considered when interpreting platform comparisons, as their relative performance may change over time. In addition, multimodal single-cell approaches have enabled the simultaneous measurement of transcriptomes and surface protein expression at single-cell resolution (29). These technologies can link transcriptome-defined granulocyte subsets to phenotypic features and functional states that have historically been inferred from surface antigen-based classifications.

## scRNA-seq approaches for revealing granulocyte heterogeneity

Since the early 2020s, scRNA-seq studies on human neutrophils have expanded rapidly. A pioneering study profiled neutrophils from the mouse bone marrow, peripheral blood, and spleen, as well as from human peripheral blood (30). Human neutrophils were subdivided into interferon (IFN) signature gene (ISG)-high, *CXCR4*-high, and *S100A12*-high subsets. The ISG-high subset was found to be transcriptionally conserved in both mice and humans. A study that applied scRNA-seq to human neutrophils from peripheral blood and bone marrow identified subsets defined by the differentiation stage and activation of IFN- or G-CSF-associated transcriptional programs (31). *Ex vivo* stimulation experiments further demonstrated that type I and type II IFNs induce distinct gene expression signatures in human neutrophils, highlighting the responsiveness of neutrophil transcriptomes to inflammatory cues. To further elucidate neutrophil heterogeneity across developmental stages, scRNA-seq has also been applied to neutrophil progenitor cells (NCPs) in the human bone marrow (32). The NCPs were subdivided into *TOP2A*-high, ISG-high, *CTSG*-high, and *BEX1*-high subsets. Neutrophil maturation is suggested to proceed along at least two differentiation trajectories: a conventional trajectory and an ISG-associated trajectory. These findings suggest that interferons influence neutrophil fate decisions during the early stages of differentiation, although the relative contributions of type I, type II, and type III interferons were not determined. Further, scRNA-seq analyses of umbilical cord blood have identified six neutrophil subsets that closely resemble those observed in adult peripheral blood (33). Among these subsets, ISG-high neutrophils were less prevalent in the cord blood. Postnatal environmental exposures may induce ISG expression in neutrophils after birth.

An important aspect that arises from these studies is the relationship between density-based neutrophil fractions and scRNA-seq–defined subsets. To address this aspect, scRNA-seq was performed on peripheral blood neutrophils separated into NDN and LDN fractions (34). These analyses revealed that NDNs largely correspond to mature neutrophils, whereas LDN populations yield a broad spectrum of differentiation states ranging from immature to mature cells. Consistent with earlier functional observations, transcriptional subsets enriched in genes associated with NET formation, including *MMP9* and *PADI4*, were preferentially represented in the LDN fraction. Collectively, scRNA-seq has revealed extensive heterogeneity among human neutrophils and provided mechanistic insights into differentiation trajectories, cytokine-induced transcriptional programs, and the molecular basis for classical neutrophil classification.

## Neutrophil heterogeneity in autoimmune and autoinflammatory diseases

Systemic lupus erythematosus (SLE) is a systemic autoimmune disorder characterized by the production of autoantibodies against DNA and the sustained activation of type I IFN pathways. Clinically, SLE manifests as heterogeneous organ involvement, including the hematological system, skin, and kidneys. A study that applied scRNA-seq to PBMCs from patients with SLE identified two transcriptionally distinct LDN subsets (35). One subset comprised mature neutrophils enriched with type I ISGs, whereas the other represented a more immature population. The proportion of the mature LDN subset was correlated with the organ damage index, suggesting that this subset may contribute to type I IFN-driven pathophysiology. This study provided transcriptome-level characterization of diversity within LDN subsets in the context of SLE.

Anti-neutrophil cytoplasmic antibody (ANCA)–associated vasculitis is characterized by the inflammation of small blood vessels. In treatment-naïve patients with microscopic polyangiitis (MPA), scRNA-seq analyses revealed the expansion of two neutrophil populations: an immature neutrophil subset and a subset characterized by the high expression of type II ISGs (36). The type II ISG-high neutrophil subset differentiated from mature neutrophils in response to combined stimulation with IFNγ and TNF. This inflammatory milieu enhanced the expression of myeloperoxidase and Fcγ receptors on the neutrophil surface, thereby promoting NET formation upon ANCA stimulation. Further, elevated serum IFNγ levels at disease onset were associated with an increased risk of relapse, supporting the clinical relevance of type II IFN-driven neutrophil programs in MPA.

In Behçet’s disease with uveitis, scRNA-seq analysis of peripheral blood neutrophils revealed sex-associated differences in the neutrophil subset composition (37). Compared with female patients, male patients exhibited a higher proportion of neutrophils expressing NETosis-related genes, such as *S100A12* and *MMP9*, whereas female patients showed relative enrichment of neutrophils with the expression of ISGs and *ANXA1*. Mechanistic analyses revealed that type I ISG-enriched neutrophils promoted regulatory T (Treg) cell induction. Neutrophil-specific deletion of *ANXA1* reduced splenic Treg cell abundance in a mouse model of uveitis. These findings indicate that sex-dependent differences in neutrophil states may contribute to the higher prevalence of uveitis in male patients with Behçet’s disease.

Recent studies have demonstrated neutrophil heterogeneity in diseased tissues. In Sweet syndrome, scRNA-seq combined with spatial transcriptomic analyses has been applied to skin biopsies obtained before and after treatment with anti-IL-36 receptor antibody (38). Lesional skin showed accumulation of neutrophils expressing high levels of NETosis-associated genes, such as *PDE4B* and *IL1RN*. Trajectory analyses suggested a blood-to-skin migratory path characterized by *PADI4* upregulation in patients with Sweet syndrome. These data suggest a pathogenic positive feedback loop involving cleavage of keratinocyte-derived IL-36 and activation by neutrophil elastase within NETs, thereby amplifying local inflammation through the reciprocal activation of keratinocytes and neutrophils.

In inflammatory bowel disease, scRNA-seq and spatial transcriptomics of colon tissues have identified three subsets of infiltrating neutrophils, designated as N1, N2, and N3, which are characterized by the high expression of *CXCL8*/*S100A9*, *CCL3*, and ISGs, respectively (39). Colon biopsy samples from patients with Crohn’s disease were enriched for N3 neutrophils, whereas samples from patients with ulcerative colitis contained a higher proportion of N1 neutrophils. Spatial analyses demonstrated the localization of these neutrophil subsets to crypt abscesses and ulcerated regions, indicating spatially organized neutrophil heterogeneity within the inflamed tissues.

Overall, scRNA-seq studies on autoimmune and autoinflammatory diseases have highlighted substantial neutrophil heterogeneity (Table 1). Among the diverse neutrophil states identified, NETosis-related and ISG-associated transcriptional programs represent shared features across different inflammatory disease contexts, both in peripheral blood and tissue microenvironments (Fig. 1).

**Figure 1.**
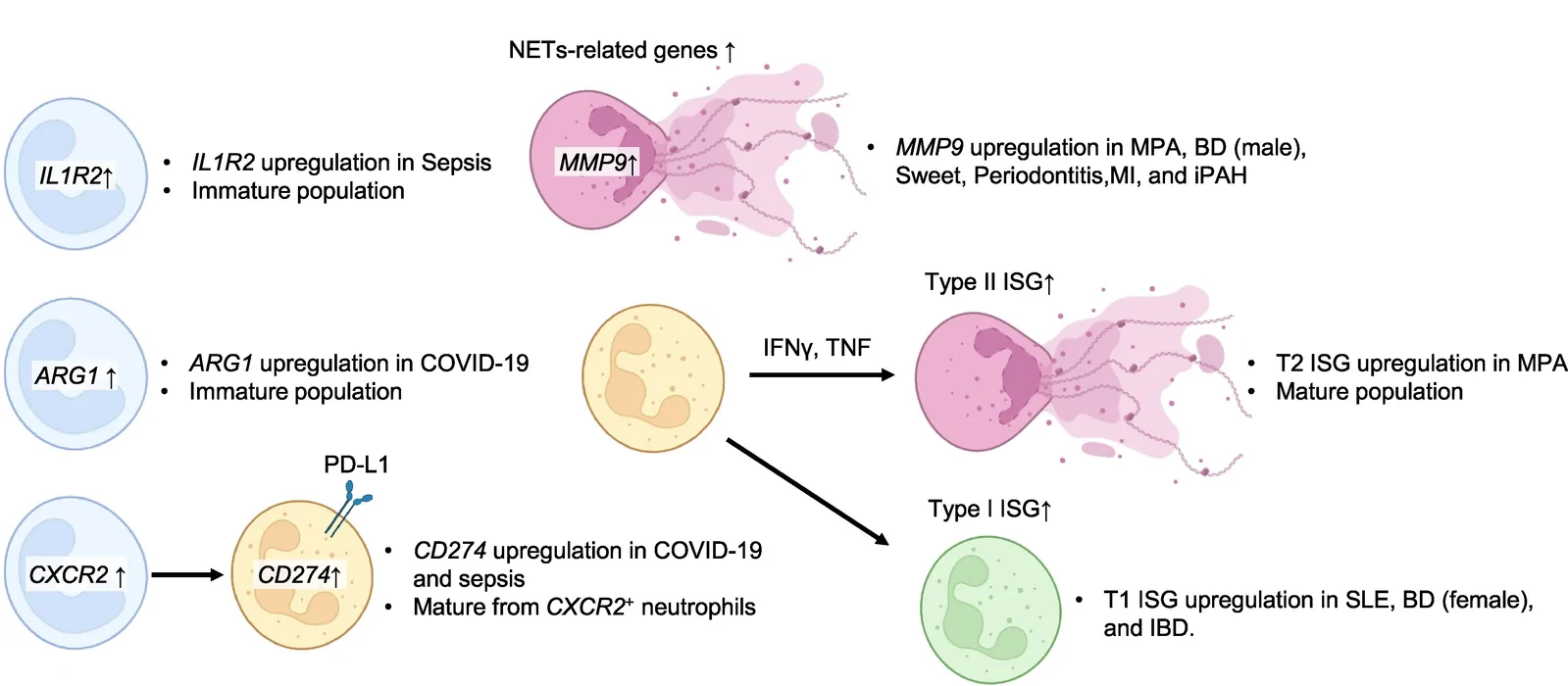
Transcriptome-based diversity of neutrophils revealed by single-cell analysis. Single-cell analyses of human neutrophils have revealed context-dependent enrichment of clinically relevant neutrophil states. Immunosuppressive neutrophil states characterized by high expression of *IL1R2*, *ARG1*, or *CXCR2* were identified in patients with infectious diseases such as sepsis and COVID-19. These cells are predominantly observed within immature populations. NET-prone neutrophils expressing *MMP9* are enriched in patients with MPA, BD, Sweet, periodontitis, MI, and iPAH, suggesting that NET formation contributes to a broad spectrum of diseases. Neutrophils with high expression of type I ISGs are enriched in patients with SLE, BD, and IBD, although their functional roles in these disease contexts remain to be fully validated. Type II ISG–high neutrophils arise from mature neutrophils in response to combined stimulation with IFNγ and TNF in patients with MPA. COVID-19, coronavirus disease 2019; NET, neutrophil extracellular trap; MPA, microscopic polyangiitis; BD, Behçet’s disease; Sweet, Sweet syndrome; MI, myocardial infarction; iPAH, idiopathic pulmonary arterial hypertension; ISG, interferon signature gene; SLE, systemic lupus erythematosus; IBD, inflammatory bowel disease. Created in BioRender. Shimagami, H. (2026) https://BioRender.com/7f2qwjd.

**Table 1.** Neutrophil and eosinophil single-cell analysis in immunological and allergic diseases.

Year	Author	Specimen	Number of donors	Modality	Platform	Key findings	Ref.
2019	Mistry P *et al.*	PB	3 SLE	scRNA-seq	10x Chromium	LDNs from patients with SLE were subdivided into a mature ISG-high subset and an immature subset.	(35)
2023	Garrido-Trigo A *et al.*	Colon	3 CD, 3 UC, 3 HD	Spatial transcriptomics	CosMX	In colon, ISG-high neutrophils were enriched in CD patients, whereas the *CXCL8*/*S100A9*-high neutrophils were enriched in UC patients.	(39)
2024	Wang Q *et al.*	PB	18 BD-BU, 16 HD	scRNA-seq	10x Chromium	Male patients with BD-BU exhibited an increase of NETs-related neutrophils. ISG-high neutrophils were abundant in female patients.	(37)
2024	Iwasaki N *et al.*	NP, PB	5 CRSwNP, 5 HD	scRNA-seq	BD Rhapsody	Inflammation and cell migration-related genes were found to be upregulated in the eosinophils of NPs from patients with CRSwNP.	(40)
2024	Liu C *et al.*	NP	Eosinophilic CRSwNP 46, non-eosinophilic CRSwNP 31	Spatial transcriptomics	GeoMX	*CCL13*-expressing *ALOX15*^+^ M2-like macrophages may contribute to epithelial remodeling in nasal polyps.	(41)
2025	Nishide M *et al.*	PB	6 MPA, 7 HD	scRNA-seq	BD Rhapsody	Type II ISG-high neutrophils were enriched in patients with MPA, and may have contributed to NETs production upon ANCA stimulation.	(36)
2025	Iwasaki N *et al.*	NP, PB	5 CRSwNP, 5 HD	scRNA-seq	BD Rhapsody	Neutrophils from patients with CRSwNP were subdivided into four subsets.	(42)
2025	Rodrigo-Munoz JM *et al.*	PB	3 asthma, 3 HD	scRNA-seq	BD Rhapsody	The composition of eosinophil subsets was similar between patients with BA and HDs, although a *S100A8*-high subset was more frequent in the BA group.	(43)
2025	Liao G *et al.*	NP, PB	6 CRSwNP, 4 CRSsNP, 5 HD. Validation cohort; 61 CRSwNP, 45 CRSsNP, 7 HD.	scRNA-seq spatial transcriptomics	10x Chromium GeoMX	*CCL13*- and *CCL18*-expressing macrophages were found to facilitate eosinophil migration toward epithelial compartments.	(44)
2026	Fu Y *et al.*	Skin, PB	7 SS, 5 HD	scRNA-seq Spatial transcriptomics	BD Rhapsody 10x Visium	NETs-related neutrophils accumulated in the skin of patients with Sweet syndrome, reflecting recruitment from the blood via a *PADI4*-high migratory axis.	(38)

Abbreviations: PB, peripheral blood; NP, nasal polyp; SLE, systemic lupus erythematosus; MPA, microscopic polyangiitis; CD, Crohn’s disease; UC, ulcerative colitis; HD, healthy donor; CRSwNP, chronic rhinosinusitis with nasal polyps; CRSsNP, chronic rhinosinusitis without nasal polyps; LDN, low-density neutrophil; NET, neutrophil extracellular trap; ISG, Interferon signature gene, BD-BU, Behçet’s disease-associated uveitis.

## Neutrophil heterogeneity in infectious diseases

During infection, neutrophils are the key effectors of both host defense and immunopathology. Recent single-cell studies have revealed that distinct transcriptional neutrophil states are linked to disease severity, tissue inflammation, and therapeutic responses. A study that applied scRNA-seq to peripheral white blood cells from patients with coronavirus disease 2019 (COVID-19) demonstrated that LDNs include both immature and mature neutrophils (45). Patients with COVID-19 showed marked expansion of immature neutrophils and ISG-enriched mature neutrophils. Functionally, neutrophils from patients with severe disease exhibited increased surface expression of PD-L1 and reduced oxidative burst capacity, suggesting that the expanded immature neutrophil population corresponds to a PMN-MDSC-like subset. Similarly, several studies have reported an expansion of neutrophil subsets expressing ISGs and inflammatory cytokines in patients with COVID-19 (46, 47). Dexamethasone treatment reduced the ISG-high neutrophil subset while increasing the *ARG1*-high immature neutrophil population with immunosuppressive feature (48). The expression levels of genes downregulated by dexamethasone treatment correlated with the clinical prognosis.

Analysis of airway samples further highlighted tissue-associated neutrophil heterogeneity. In patients with COVID-19, scRNA-seq analyses of endotracheal aspirates and peripheral white blood cells suggested that circulating *IFITM2*-high neutrophils are recruited to the site of inflammation via the CXCL8–CXCR2 axis and subsequently reprogrammed into an inflammatory state (49). In patients with COVID-19–associated pulmonary aspergillosis (CAPA), the proportion of neutrophils in the bronchoalveolar lavage fluid (BALF) was reduced, and the remaining BALF neutrophils were skewed toward an HLA class II-associated transcriptional state (50). Notably, the concentration of H3Cit-DNA, a PAD4-dependent NETosis biomarker, was elevated in the BALF of patients with CAPA. Higher H3Cit-DNA levels were associated with improved 90-day survival, suggesting that increased NET formation may partly account for the reduced neutrophil proportion and reflect an effective antifungal host response rather than purely tissue-damaging inflammation.

In bacterial sepsis, scRNA-seq studies have revealed the expansion of an *IL1R2*-high immature neutrophil subset with immunosuppressive properties (51). In co-culture assays, neutrophils derived from patients with sepsis suppressed CD4^+^ T cell proliferation and activation. As effective CD4^+^ T cell responses are critical for resolving infection, expansion of this immunosuppressive neutrophil subset may contribute to the progression of bacterial infection. In another study, *CXCR2*-high neutrophils were shown to differentiate into *CD274*-high immunosuppressive neutrophils (52). The CXCR2 antagonist SB225002 selectively targeted these immunosuppressive neutrophil subsets and improved outcomes in a mouse model of sepsis. In contrast, the expansion of *MMP9*/*S100A9*-high neutrophils is associated with progression to septic shock in patients with bacterial infection (53). These findings suggest that distinct neutrophil subsets contribute to different aspects of sepsis pathophysiology. scRNA-seq of gingival tissue from patients with periodontitis revealed accumulation of neutrophils with an activated NET formation program (54). DNase I treatment ameliorated disease severity in a mouse model of periodontitis. DNase I reduced CitH3 protein levels and inflammatory cell infiltration in gingival tissues, supporting the pathogenic role of NETs in periodontal inflammation. Cell–cell interaction analyses further indicated that macrophage migration inhibitory factor (MIF) produced by gingival fibroblasts promoted neutrophil recruitment, highlighting MIF as a potential predictive marker and therapeutic target in periodontitis.

In summary, single-cell profiling revealed substantial heterogeneity among neutrophil subsets in infectious diseases (Table 2). NETosis-associated and ISG-high neutrophil states are associated with inflammatory pathology, whereas immature neutrophil subsets expressing *IL1R2*, *ARG1*, or *CXCR2* exhibit immunoregulatory features. The expansion of such immunosuppressive neutrophil populations may contribute to the susceptibility to secondary infections and represent a potential target for prognostic stratification and therapeutic intervention.

**Table 2.** Neutrophil single-cell analysis in infectious diseases.

Year	Author	Specimen	Number of donors	Modality	Platform	Key findings	Ref.
2020	Schulte-Schrepping J *et al.*	PB	18 COVID-19, 16 HD	scRNA-seq	BD Rhapsody	A PMN-MDSC-like, immature neutrophil subset was enriched in patients with severe COVID-19.	(45)
2022	Sinha S *et al.*	PB	8 COVID-19 ARDS, 6 COVID-19 ARDS with DEX, 6 bacterial ARDS, 5 HD	scRNA-seq	10x Chromium	Dexamethasone treatment increased the *ARG1*-high immature neutrophil subset with immunosuppressive features.	(48)
2022	Xu J *et al.*	PB	6 severe, 4 mild COVID-19, 5 HD	scRNA-seq	10x Chromium	Severe COVID-19 was associated with an increase of neutrophil subsets characterized by NF-κB signaling and oxidative metabolism.	(46)
2022	Hong Y *et al.*	PB	12 sepsis	scRNA-seq	GEX SCOPE	Expansion of the *MMP9*-high neutrophil subset was associated with an increased risk of septic shock in patients with sepsis.	(53)
2023	Eddins DJ *et al.*	PB	18 severe, 9 mild COVID-19, 8 HD	scRNA-seq	10x Chromium	Mature neutrophils accumulated in the airways of patients with COVID-19 via CXCL8-CXCR2 axis and were reprogrammed into an inflammatory state.	(49)
2023	Kwok AJ *et al.*	PB	26 sepsis, 6 HD, 7 sterile inflammations	scRNA-seq	BD Rhapsody	*IL1R2*-high immature neutrophil subset was associated with sepsis exacerbation.	(51)
2024	Feys S *et al.*	BALF	22 COVID-19 only, 14 CAPA	scRNA-seq	10x Chromium	HLA class II-associated neutrophil subset was increased in patients with CAPA, accompanied by elevated NET formation.	(50)
2025	Shen R *et al.*	BALF	4 sepsis	scRNA-seq	10x Chromium	*CXCR2*-high neutrophils in the BALF of sepsis patients might differentiate into a *CD274*-high subset, contribute to sepsis-associated immunosuppression.	(52)
2025	Qiu W *et al.*	Gingiva	3 Periodontitis, 3 HD	scRNA-seq	BD Rhapsody	Gingival fibroblast-derived MIF might promote the migration of NETs-related neutrophil subset, and NETs contribute to periodontitis.	(54)

Abbreviations: PB, peripheral blood; BALF, bronchoalveolar lavage fluid; COVID-19, coronavirus disease 2019; HD, healthy donor; ARDS, acute respiratory distress syndrome; DEX, dexamethasone; CAPA, COVID-19–associated pulmonary aspergillosis; PMN-MDSC, polymorphonuclear myeloid-derived suppressor cells; NET, neutrophil extracellular trap; MIF, migration inhibitory factor; HLA, human leukocyte antigen.

## Neutrophil heterogeneity in cardiovascular diseases

Cardiovascular diseases are recognized as conditions shaped by hemodynamic and metabolic factors as well as by local immune responses within the vascular system. Among immune cells, granulocytes play critical roles in vascular inflammation, ischemia/reperfusion injury, and thrombosis (55, 56). In patients with myocardial infarction (MI), scRNA-seq of peripheral neutrophils identified the expansion of a neutrophil subset characterized by high expression of NETosis-related genes, including *MMP9* (57). Consistent with these findings, peripheral neutrophils from patients with MI exhibit enhanced NET formation compared with those from healthy donors. Previous studies showed that NETs exacerbate myocardial ischemia–reperfusion injury by promoting microvascular obstruction, inflammation, and thrombosis in mouse models (58). Collectively, these observations suggest that NET-prone neutrophils may contribute to post-ischemic myocardial injury following MI. Furthermore, scRNA-seq analysis of coronary thrombi from patients with MI revealed that intrathrombotic neutrophils strongly expressed inflammation-related genes, whereas neutrophil subsets exhibiting exhausted transcriptional signatures were markedly downregulated (5).

Peripheral blood white blood cells from patients with deep vein thrombosis (DVT) were analyzed using both bulk RNA-seq and scRNA-seq (59). Network analysis of the transcriptomic data revealed several pattern-recognition receptors, including *DDX58*, *TLR1*, and *TLR8*, which were highly expressed in neutrophils, suggesting that neutrophil sensing of danger-associated signals may contribute to the pathogenesis of DVT. Peripheral blood neutrophils from patients with idiopathic pulmonary arterial hypertension (IPAH) were profiled using scRNA-seq and classified into five transcriptionally distinct subsets (60). Patients with a high proportion of *MMP9*-high neutrophils exhibited poorer clinical outcomes, suggesting that this subset may serve as a prognostic indicator of IPAH.

Collectively, accumulating evidence has linked neutrophil heterogeneity to cardiovascular diseases. In particular, an *MMP9*-positive NET-prone neutrophil subset has emerged across multiple cardiovascular contexts as a potential prognostic biomarker.

## Eosinophil heterogeneity in allergic diseases

In allergic diseases, increasing attention has been directed toward the heterogeneity of eosinophils, particularly in chronic rhinosinusitis with nasal polyps (CRSwNP). CRSwNP is a chronic inflammatory airway disease characterized by persistent sinonasal inflammation, nasal polyp formation, and prominent eosinophilic infiltration of the affected tissues (61). Granulocytes isolated from the nasal polyps of patients with CRSwNP were analyzed using scRNA-seq (40, 42). Eosinophils within diseased polyps showed high expression of genes involved in inflammation and cell migration, whereas peripheral blood eosinophils from patients showed only modest transcriptomic differences compared with those from healthy controls. Similarly, scRNA-seq analyses of peripheral blood eosinophils from patients with bronchial asthma did not reveal major differences in the eosinophil subset composition compared with those from the healthy controls (43).

Despite minimal transcriptional changes in circulating eosinophils, they accumulate abundantly within nasal polyps, suggesting that alterations in other tissue-resident cells contribute to disease pathology. Spatial transcriptomic analysis of nasal polyps from patients with CRSwNP revealed high expression of genes associated with epithelial remodeling and epithelial–mesenchymal transition (EMT) within the epithelial compartment (41). Integration of spatial transcriptomics with scRNA-seq further confirmed the accumulation of *ALOX15*-positive M2-like macrophages expressing high levels of *CCL13*. Transcriptional changes in epithelial cells correlated with the abundance of these macrophages, and CCL13 blockade reduced EMT in primary human nasal epithelial cells co-cultured with macrophages, supporting the role of macrophage-derived CCL13 in epithelial remodeling within nasal polyps. Further, a study that combined spatial transcriptomics and scRNA-seq reported that *CCL13*- and *CCL18*-expressing macrophages localized to epithelial regions in the nasal polyps of patients with CRSwNP. These macrophages produce chemokines that facilitate eosinophil migration toward the epithelial compartments (44).

Currently, the scRNA-based analysis of eosinophils has several technical limitations. Although the detailed profiling of tissue-infiltrating eosinophils is critical for understanding the roles of eosinophil subsets in allergic diseases, most transcriptome-defined eosinophil subsets are not linked to specific functional properties. Tissue eosinophils are often not robustly captured by scRNA-seq, likely because of cell loss during tissue dissociation. These limitations suggest that spatial transcriptomics may be more suitable than scRNA-seq for detecting tissue-resident eosinophils. Further technical advances and close bed-to-bench collaboration to preserve cell quality during tissue processing are required to enable high-resolution analysis of tissue-resident eosinophil heterogeneity and their interactions with the surrounding tissue cells in allergic diseases.

## Conclusion and future perspectives

As summarized in this review, scRNA-seq studies have revealed diverse neutrophil states across multiple diseases. The expansion of NETosis-associated and ISG-high neutrophil subsets has been linked to immunological, infectious, and inflammatory disease processes and is often correlated with clinical outcomes. In MPA, serum IFNγ levels at disease onset may serve as a surrogate marker of specific neutrophil population characterized by transcriptomic changes and may help predict subsequent disease relapse. In COVID-19 and sepsis, *ARG1*- and *IL1R2*-expressing neutrophils have the potential to predict clinical outcomes, while the identification of clinically applicable surrogate markers for these neutrophil populations still needs to be established. Importantly, these granulocyte states are difficult to define by using cell surface marker-based analyses, demonstrating the impact of single-cell transcriptome technologies. Despite these technical advances, several key challenges remain.

First, as a general limitation of scRNA-seq–based classification, the number and identity of inferred cell clusters can be influenced by analytic choices, including batch correction, feature selection, dimensionality reduction, clustering resolution, and annotation strategies. In granulocyte studies, this limitation is further complicated by the lack of a consensus framework for defining granulocyte subsets, which complicates data integration and comparison across studies.

Similarly labeled populations, such as “NETosis-associated,” “ISG-high,” or “immature” neutrophils, have been reported across disease states; however, whether these designations reflect shared immune abnormalities or distinct disease-specific states remains unclear. Subtle but biologically meaningful differences may be overlooked, even within identically labeled populations (24). Therefore, standardized analytical approaches, code sharing, and the establishment of a community-wide standardized nomenclature will be essential (62). Recent efforts involving open data sharing and large-scale single-cell datasets of granulocytes have been implemented in murine systems (63). Similar approaches in human granulocytes are required for enabling clinical translation. Second, the functional properties and maturation states of transcriptome-defined granulocyte subsets are often difficult to validate experimentally. Further, appropriate surface marker panels for isolating individual subsets remain limited. Granulocytes are short-lived and readily undergo transcriptional changes during isolation. These constraints complicate the reproducible functional validation of subset-specific properties inferred from transcriptomic data. Granulocyte-specific gene knockout animal models or engineered cell lines are needed to directly assess subset-specific functions. Third, the high-resolution profiling of granulocyte transcriptomes in affected tissues remains technically challenging. Compared with PBMCs, granulocytes contain less RNA and exhibit higher endogenous RNase activity, leading to rapid mRNA degradation during tissue dissociation (24, 64). Optimized tissue processing and preservation protocols are required. In current settings, spatial transcriptomics may be more suitable than scRNA-seq for detecting tissue granulocytes without extensive tissue homogenization (44).

In conclusion, granulocyte subsets identified using scRNA-seq are increasingly implicated in disease mechanisms and clinical outcomes. The establishment of a consensus nomenclature supported by large-scale integrative scRNA-seq analyses across diseases is awaited. Further, in-depth profiling of granulocyte subsets with the experimental validation will facilitate the development of novel biomarkers and therapeutic targets for immune-mediated and inflammatory diseases.
